# Clinical Microbiology in Xylazine-Associated Wound Infections

**DOI:** 10.1093/ofid/ofaf384

**Published:** 2025-08-07

**Authors:** Deanna M Berg, Amanda Binkley, Sean D Foster, Matthew Hinton, Derek Peiffer, Samantha C Huo, Jessie Torgersen, Christina Maguire

**Affiliations:** Department of Pharmacy Services, Penn Presbyterian Medical Center, Philadelphia, Pennsylvania, USA; Department of Pharmacy Services, Penn Presbyterian Medical Center, Philadelphia, Pennsylvania, USA; Department of Emergency Medicine, Penn Presbyterian Medical Center, Philadelphia, Pennsylvania, USA; Department of Pharmacy Services, Penn Presbyterian Medical Center, Philadelphia, Pennsylvania, USA; Department of Pharmacy Services, Penn Presbyterian Medical Center, Philadelphia, Pennsylvania, USA; Department of Emergency Medicine, Penn Presbyterian Medical Center, Philadelphia, Pennsylvania, USA; Division of Infectious Diseases, Perelman School of Medicine, University of Pennsylvania, Philadelphia, Pennsylvania, USA; Department of Biostatistics, Epidemiology, and Informatics, Center for Clinical Epidemiology and Biostatistics, University of Pennsylvania Perelman School of Medicine, Philadelphia, Pennsylvania, USA; Department of Pharmacy Services, Penn Presbyterian Medical Center, Philadelphia, Pennsylvania, USA

**Keywords:** antibiotics, antimicrobial stewardship, antimicrobials, bone and joint infections, MRSA

## Abstract

**Background:**

Injection drug use (IDU) containing xylazine has been associated with the development of chronic necrotic wounds, albeit not all are of infectious etiology. This study describes the clinical microbiology of xylazine-associated wound infections to guide antimicrobial prescribing.

**Methods:**

We conducted a retrospective, single-center cohort study of adults hospitalized with xylazine-associated wound infections related to IDU from 1 April 2022 to 1 December 2023. Patients were included if they received antimicrobials for suspected wound infections, along with either a positive urine xylazine test or urine fentanyl test with patient-reported xylazine use. Microbiology results of wound and blood cultures were collected. Antimicrobial spectrum and hospital outcomes were described, including multidrug-resistant organisms’ isolation, duration of therapy, in-hospital mortality, and 30-day readmission.

**Results:**

A total of 166 patients were included, of whom 81 had wound cultures and 153 had blood cultures collected; more than half had positive cultures (93/166 [56.0%]). Most wound cultures were positive (78/81 [96.3%]), compared to 25.5% of blood cultures (39/153). Approximately 40% of wound cultures were obtained operatively. Gram-positive organisms were isolated in nearly all wound (73/78 [93.6%]) and blood (37/39 [94.9%]) cultures. The predominant causative organisms were methicillin-resistant *Staphylococcus aureus* (MRSA) (52/93 [55.9%]) and β-hemolytic streptococci (34/93 [36.6%]). *Pseudomonas aeruginosa* was isolated in wound cultures of 8 patients, 7 with bone and joint infections.

**Conclusions:**

Empiric antimicrobial coverage for suspected xylazine-associated wound infections should include MRSA and β-hemolytic streptococci coverage. Empiric gram-negative and anaerobic coverage may be warranted for those with higher suspicion of bone and joint involvement.

## Graphical Abstract

**Graphical Abstract:**
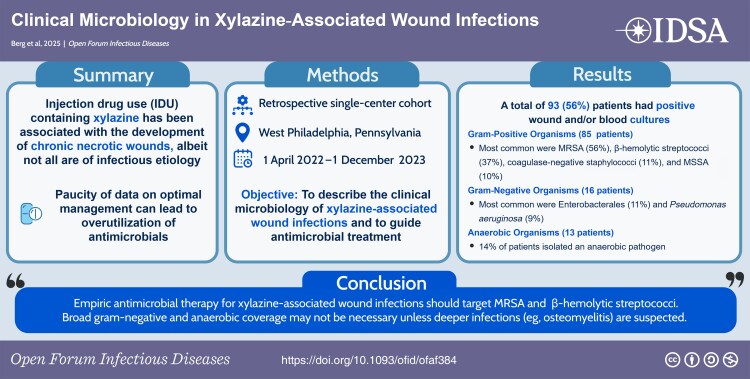
This graphical abstract is also available at Tidbit: https://tidbitapp.io/tidbits/clinical-microbiology-in-xylazine-associated-wound-infections-907b4f6c-5e1a-420d-8ed5-b6676354fd3c?utm_campaign=tidbitlinkshare&amp;utm_source=ITP

Philadelphia has one of the highest rates of morbidity related to drug overdose in the United States, and it is estimated that approximately 68 000 persons inject drugs within the city [1]. In 2021, forensic reports demonstrated that 90% of street opioids in Philadelphia contained xylazine in varying quantities (19%–45%) to enhance/prolong the effects of the opioid [2, 3]. Xylazine is a potent centrally acting α-2 adrenergic agonist first synthesized in 1962 to be explored as a potential sedative, hypnotic, anesthetic, and analgesic [4]. Xylazine use is restricted as a veterinarian sedative, given its risk for profound and sustained hypotension [4, 5].

An additional side effect of xylazine use includes necrotic skin wounds [6, 7]. The majority of xylazine-associated wounds develop at the injection site. However, lesions have been observed at noninjection sites and with other routes of administration (eg, intranasal, inhalation) [8]. Xylazine-associated skin necrosis is likely attributed to the activation of α-1- and α-2 adrenergic receptors in the peripheral vasculature, resulting in vasoconstriction and insufficient tissue perfusion [4, 5, 9]. Local infiltration into the subcutaneous tissue or muscle can delay absorption, prolonging deprivation of adequate tissue perfusion and necrosis. Repetitive use compromises wound healing, thus increasing the likelihood of the formation of chronic necrotic wounds [7, 9]. To determine if a chronic wound is infected, clinicians look for evidence of acute changes in the wound appearance (eg, increased erythema at edges), increased pain or drainage, physical examination findings, or laboratory data suggesting the presence of an acute bacterial process. Infected xylazine-associated wounds have been previously described by McFadden et al [7].

Limited data are available to inform best practices for the management of xylazine-associated wound infections. Optimal wound management, including the need for broad antimicrobial coverage, remains to be determined. This study aims to describe the culture positivity and clinical microbiology of xylazine-associated wound infections to inform optimal antimicrobial coverage.

## METHODS

### Study Design

We conducted a retrospective, single-center, cohort study of adults with xylazine-associated wound infections related to injection drug use (IDU) at Penn Presbyterian Medical Center in West Philadelphia, Pennsylvania, admitted between 1 April 2022 and 1 December 2023.

### Study Population

EPIC Reporting Workbench, a tool within the electronic health record (EHR), was utilized to identify patients. Screening of adult patients (aged ≥18 years) with active IDU occurred in 2 stages: (1) All patients with an order for a urine xylazine confirmation test were screened first; (2) patients with a positive urine fentanyl test with an encounter diagnosis suggestive of a potential xylazine-associated wound infection (eg, 1 or more *International Classification of Diseases, Tenth Revision* diagnostic codes related to cellulitis, cutaneous abscess, wound infection, sepsis, bacteremia, osteomyelitis, IDU, etc, recorded during hospitalization) were then screened. Patients were only included in the study once; if a patient had multiple hospitalizations and/or wound cultures, the earliest encounter with the single most invasive method of wound culture acquisition was utilized. All cultures (ie, blood and wound) were collected from the same encounter. Screening concluded once all patients in the preliminary screening were reviewed to include a maximum of 200 patients as a sample size of convenience. The primary author conducted the chart review. Other members of the research team were consulted to review cases as needed. Patients were included if they received antimicrobial treatment for a suspected wound infection related to IDU and had documentation of either a positive urine xylazine confirmation test or patient-reported xylazine use with a positive urine fentanyl test during the encounter of interest. Additional details on the inclusion criteria are expanded upon in the Supplementary Appendix. Patients were excluded if the treating provider did not suspect the xylazine-associated wound was infected or the patient did not receive antimicrobial therapy for xylazine-associated wounds.

### Patient Consent Statement

The Institutional Review Board at the University of Pennsylvania reviewed and approved the study. Owing to the retrospective nature of this study, a waiver to obtain informed consent and Health Insurance Portability and Accountability Act authorization was granted.

### Outcomes

The primary outcome was to define the culture positivity rate and clinical microbiology of xylazine-associated wounds. The primary endpoints included reporting culture positivity, and all organisms isolated in wound or blood cultures. Blood cultures were included as wounds can serve as portal of entry for subsequent bacteremia. The secondary outcomes included describing the empiric antimicrobial spectrum coverage (ie, gram-positive, gram-negative, anaerobic) and IDU practices (ie, licking needles prior to injection, reusing or sharing needles, or rinsing with water). Additionally, rates of multidrug-resistant organism (MDRO) isolation, duration of antimicrobial therapy, in-hospital mortality, and 30-day readmission were reported. Diagnosis of suspected or invasive infection was determined based on documentation of bone or joint infection (BJI) by the treating provider or presence of bacteremia based on positive blood cultures. Housing instability was determined based on documentation in the EHR by any medical provider indicating the patient was actively experiencing housing instability. Additional definitions are described in the Supplementary Appendix.

### Data Collection

Demographic, clinical, and microbiological data were extracted from the EHR. The extracted data were anonymized and entered into Penn Medicine's REDCap (Research Electronic Data Capture). BD BACTEC FX (BD Diagnostics) was used for rapid detection of bacteria and fungi in blood cultures. Matrix-assisted laser desorption/ionization–time-of-flight mass spectrometry (Bruker Corporation) and VITEK MS (bioMérieux) were used for microbial identification.

### Statistical Analysis

Study variables were assessed using descriptive statistics, including frequencies and percentages for categorical variables and median with interquartile range (IQR) or mean with standard deviation for continuous variables.

## RESULTS

### Patient Demographics

Of the 685 patients with positive urine toxicology screened, 166 met the inclusion criteria. Characteristics of the included patients are detailed in Table 1. The majority were assigned male sex at birth (68.1%) with a median age of 39 years (IQR, 33–45 years), had recent antimicrobial therapy (55.4%) and experiencing housing instability (68.1%). Limited data were documented on IDU practices: 23 (13.9%) patients reported licking needles prior to injection, reusing or sharing needles, or rinsing with water (data not shown).

**Table 1.** Characteristics of Patients With Suspected Xylazine-Associated Wound Infections

Characteristic	Total (N = 166)
Age, y, median (IQR)	39 (33–45)
Male sex assigned at birth	113 (68)
Housing instability	113 (68)
History within the past 90 d	
Xylazine-associated wounds^a^	90 (54)
Hospitalization for at least 48 h	71 (43)
ED visits or hospitalizations, median (IQR)	1 (0–4)
Antimicrobial use for at least 48 h^b^	92 (55)
Positive urine xylazine assay	34 (21)
Positive within the past 12 mo	8 (5)
Terminology used to describe xylazine^c^	
Xylazine	104 (63)
Tranq	66 (40)
Tranquilizer	11 (7)
Kensington Dope or Dope from Kensington	2 (1)
Tranq-dope	2 (1)
Method of wound culture collection	
Bedside	49 (30)
Operative	32 (19)
Suspected or confirmed invasive infection^d^	60 (36)
Bone or joint infection	44 (27)
Bacteremia	36 (22)

Data are reported as No. (%) unless otherwise indicated.

Abbreviations: ED, emergency department; IQR, interquartile range.

^a^Xylazine-associated wounds that required antimicrobial treatment.

^b^Antimicrobial use for any indication.

^c^Patient-reported during encounter of interest.

^d^Diagnosis of suspected or invasive infection was determined based on documentation of bone or joint infection by the treating provider or presence of bacteremia based on positive blood cultures.

### Empiric Antimicrobial Therapy

Nearly all patients (99.4%) received empiric coverage against methicillin-resistant *Staphylococcus aureus* (MRSA). More than half of the patients (57.2%) received empiric gram-negative coverage; notably, 81 (48.8%) included anti-pseudomonal coverage. Fifty-seven patients received anaerobic coverage (34.3%) (Supplementary Table 1). Empiric coverage against extended-spectrum β-lactamase–producing organisms was not utilized for any patient.

### Microbiological Data

Ninety-three (56.0%) patients had positive cultures, including 78 patients with positive wound cultures and 39 patients with positive blood cultures, totaling 117 cultures with microbiological results. Wound cultures were positive in 96.3% (78/81) of individuals, and 39.5% of those wound cultures were obtained during operative intervention (Table 2). Blood cultures were positive in 25.5% (39/153) of individuals. Of the positive wound and blood cultures, nearly all cultures (91.4%) isolated a gram-positive organism. MRSA (52/93 [55.9%]) and β-hemolytic streptococci (34/93 [36.6%]) were the most common pathogens.

**Table 2.** Wound and Blood Culture Results From Patients With Xylazine-Associated Wounds

Variable	Wound Cultures			Blood	Total
	Bedside/Operative	Bedside	Operative^a^		
All patients	n = 81	n = 49	n = 32	n = 153	n = 166
Culture negative	3 (4)	1 (2)	2 (6)	114 (75)	73 (44)
Culture positive	78 (96)	48 (98)	30 (94)	39 (25)	93 (56)
Polymicrobial	30 (37)	14 (29)	16 (50)	4 (3)	32 (19)
Patients with positive cultures^b^	n = 78	n = 48	n = 30	n = 39	n = 93
Gram-positive organism	73 (94)	46 (96)	27 (90)	37 (95)	85 (91)
MRSA	46 (59)	31 (65)	15 (50)	16 (41)	52 (56)
β-hemolytic streptococci	24 (31)	15 (31)	9 (30)	12 (31)	34 (37)
CoNS	7 (9)	5 (10)	2 (7)	3 (8)	10 (11)
MSSA	5 (6)	2 (4)	3 (10)	6 (15)	9 (10)
*Enterococcus* species^c^	5 (6)	…	5 (17)	…	5 (5)
Other^d^	3 (4)	1 (2)	2 (7)	1 (3)	4 (4)
Gram-negative organism	16 (21)	6 (13)	10 (33)	1 (3)	16 (17)
Enterobacterales	10 (13)	5 (10)	5 (17)	1 (3)	10 (11)
*Pseudomonas aeruginosa*	8 (10)	1 (2)	7 (23)	…	8 (9)
Other^e^	4 (5)	1 (2)	3 (10)	…	4 (4)
Anaerobic organism	11 (14)	3 (6)	8 (27)	2 (5)	13 (14)
Gram-negative anaerobe	6 (8)	…	6 (20)	1 (3)	7 (8)
Gram-positive anaerobe	5 (6)	3 (6)	2 (7)	1 (3)	6 (7)
*Candida* species	1 (1)	…	1 (3)	1 (3)	2 (2)

Data are reported as No. (%).

Abbreviations: CoNS, coagulase-negative staphylococci; MRSA, methicillin-resistant *Staphylococcus aureus*; MSSA, methicillin-susceptible *Staphylococcus aureus*.

^a^Operative cultures were defined as any aseptically obtained culture in the operating room or interventional radiology.

^b^Organisms recovered are not mutually exclusive.

^c^ *Enterococcus* species included 3 *Enterococcus faecalis* isolates, 1 *Enterococcus faecium*, and 1 *Enterococcus gallinarum*.

^d^Other gram-positive species included 1 viridians group streptococci isolate from a bedside culture, 1 *Cutibacterium* species isolate from an operative culture, 1 unidentifiable isolate from an operative culture, and 1 *Corynebacterium* species from a blood culture.

^e^Other gram-negative species included 1 *Stenotrophomonas maltophilia* isolate from an operative culture, 1 *Alcaligenes faecalis* isolate from an operative culture, 1 *Bordetella trematum* isolate from an operative culture, and 1 unidentifiable isolate from a bedside culture.

When looking specifically at wound cultures, the most common pathogens remained *S aureus* (51/78 [65.4%]) and β-hemolytic streptococci (24/78 [30.8%]), while Enterobacterales were not routinely observed (10/78 [12.8%]). Most *S aureus* isolates were methicillin-resistant (46/51 [90.2%]) (Table 2). Supplementary Table 2 describes isolated Enterobacterales species. Of the 10 patients who isolated Enterobacterales, 6 had confirmed or suspected BJI. *Pseudomonas aeruginosa* was isolated in 8 wound cultures, of which 6 were polymicrobial with a gram-positive pathogen (Table 3). Confirmed or suspected BJI was present in 44 patients, of whom 30 had positive culture data. Of those patients, 7 isolated *P aeruginosa*.

**Table 3.** Patients With Isolated *Pseudomonas aeruginosa* in Xylazine-Associated Wound Cultures

Characteristic	Total (n = 8)
Confirmed or suspected invasive infection^a^	8 (100)
Bone or joint infection	7 (88)
Bacteremia	5 (63)
History within the past 90 d	
Hospitalization for at least 48 h	5 (63)
ED visits or hospitalizations, median (IQR)	3 (1–3)
Antimicrobial use for at least 48 h	6 (75)
Xylazine-associated wound infections	6 (75)
Housing instability	3 (38)

Data are reported as No. (%) unless otherwise indicated. None of the patients isolated *Pseudomonas aeruginosa* in blood cultures.

Abbreviations: ED, emergency department; IQR, interquartile range.

^a^Diagnoses are not mutually exclusive.

Positive blood cultures largely isolated a gram-positive organism (37/39 [94.9%]) (Table 2). MRSA bacteremia was identified in 16 patients. The isolation of anaerobic bacteria and Enterobacterales species occurred in 2 patients and 1 patient, respectively. *Pseudomonas aeruginosa* was not isolated from blood culture for any patients. All patients with positive blood cultures also had positive wound cultures. Of the patients with bacteremia, 35.9% (14/39) isolated the same organism in blood and wound cultures (Supplementary Table 3).

### Clinical Course

Due to the frequency of patient-directed discharge, not all patients were able to undergo or complete confirmatory testing for invasive infection (eg, blood cultures, radiographic imaging). Among the patients with confirmed or suspected BJI, 16 of 44 patients elected to undergo surgical debridement. None of the patients underwent surgical amputation. One in 10 patients required intensive care unit level of care during their hospitalization (Table 4). The median hospital length of stay was 5 days (IQR, 2–11 days). Patient-directed discharge was the most common hospital disposition. Two of the 91 patients discharged on antimicrobial therapy received a long-acting lipoglycopeptide. The median total duration of treatment, including outpatient therapy, was 11 days (IQR, 7–16 days). One patient with a polymicrobial bedside wound culture and MRSA bacteremia experienced in-hospital mortality 6 days after admission, unrelated to xylazine-associated wounds. Forty-five (27.1%) patients were rehospitalized for xylazine-associated wounds or undifferentiated sepsis within 30 days of discharge.

**Table 4.** Outcomes in Patients Treated for Xylazine-Associated Wound Infections

Characteristic	Total (n = 166)
Surgical debridement of wounds	27 (16)
Intensive care unit admission	17 (10)
Hospital length of stay, d, median (IQR)	5 (2–11)
Total duration of antimicrobial therapy, d, median (IQR)	11 (7–16)
Inpatient therapy	5 (2–10)
Outpatient therapy	8 (6–10)
Discharged on antimicrobial therapy	91 (55)
Hospital disposition	
Patient-directed discharge	81 (49)
Home or self-care (routine)	55 (33)
Rehabilitation facility	30 (18)
30-d rehospitalization for xylazine-associated wound infections or undifferentiated sepsis	45 (27)

Data are reported as No. (%) unless otherwise indicated.

Abbreviation: IQR, interquartile range.

## DISCUSSION

To our knowledge, this is the first study to date to highlight microbiological data in xylazine-associated wounds. Our microbiological data reflect that in the setting of xylazine-associated wounds, gram-positive organisms, namely *S aureus* and β-hemolytic streptococci, are largely isolated, while Enterobacterales and *P aeruginosa* are responsible for <20% of isolated organisms. Gram-negative isolation was primarily associated with operatively obtained cultures in invasive infections.

The Infectious Diseases Society of America guidelines for skin and soft tissue infections (SSTIs) note that IDU is a risk factor for polymicrobial infections and recommend only empiric MRSA and streptococcal coverage for the treatment of cellulitis or erysipelas [10]. Data suggest that in addition to commensal skin organisms, IDU may also be at risk for other gram-negative and anaerobic organisms due to the introduction of contaminated needles, behavioral practices (eg, licking needles, rinsing with tap water), the quality and type of drug injected, and environmental factors such as homelessness [11, 12]. This has led to many clinicians prescribing empiric gram-positive and gram-negative coverage for IDU-related SSTI.

A retrospective cohort study by Kelly et al of 230 persons who inject drugs reported gram-negative bacilli and gram-positive infection incidence rates of 28% and 72%, respectively [13]. Of the patients with risk factors for *P aeruginosa* (ie, age >50 years, prior hospitalization within 90 days, and osteoarticular infections associated with gram-negative infections), only 5% of the isolated organisms were *P aeruginosa* [13].

Our findings are that gram-positive organisms are the predominant pathogen isolated and that gram-negative organism recovery is largely limited to BJI in xylazine-associated wound infections, which echoes previous related SSTI IDU literature [11–13]. Notably, more than half of positive cultures isolated MRSA regardless of how they were obtained, closely followed by β-hemolytic streptococci [12–14]. Gram-negative organisms, specifically *P aeruginosa*, were not commonly isolated from cultures. In light of these findings, there was a large discrepancy in antimicrobial prescribing patterns compared to organisms isolated. Almost all patients received empiric MRSA coverage, while nearly half received empiric anti-pseudomonal coverage. Our data support the use of solely gram-positive coverage for noninvasive xylazine-associated wound infections while reserving additional gram-negative coverage when there is clinical concern for BJI underlying these wound infections. *Staphylococcus aureus* susceptibility data have been previously reported by Dickinson et al [15].

It is critical to note that despite appearance, not all xylazine-associated wounds have an infectious etiology. With repetitive IDU, hyperpigmentation, scarring, nodules, and eschar often develop, making wounds appear infectious [11]. Xylazine-associated wounds, in particular, create round ulcerations that develop into progressive tissue loss and necrosis that may raise alarm to clinicians [11]. Approximately half of the patients in our study who received empiric antimicrobials had negative culture data, suggesting further need for internal guidance on the management of xylazine-associated wounds to discern between infection and chronic noninfected wounds. Given the chronicity of these wounds, these patients generally present clinically stable despite the appearance of an infected, necrotic wound; thus, empiric *Pseudomonas* coverage in the absence of culture data may not provide additional benefit to the treatment of these wounds. Delaying the initiation of gram-negative coverage prior to operative cultures can increase the culture yield and increase the chances of isolating a causative pathogen.

This study has several limitations that are worth noting. Given the retrospective nature of this study, the data are subject to recall bias based on the reliance on others for record-keeping. The absence of documented patient-reported xylazine use may have resulted in the exclusion of patients and, consequently, leading to the omission of valuable culture data. Information from outside hospitals was limited to the hospital records accessible within the health information exchange function of the EHR at the time of chart review, thus potentially underestimating the rate of healthcare exposure, history of MDRO, and rehospitalizations. If multiple encounters were eligible for inclusion, the decision was made to utilize the patient's encounter with the most invasive culture in an effort to increase the accuracy of identifying the causative organism. While our findings can inform empiric antimicrobial coverage for xylazine-associated wound infections based on recovered microbes, complete clinical evaluation and severity of illness should drive patient-level treatment decisions. The method of culture collection should be carefully scrutinized as bedside swabs are more susceptible to contamination, whereas operative cultures obtained through invasive procedures provide a more reliable microbiological assessment. The determination of an infected versus chronic wound was at the discretion of the treating clinician. Cultures of chronic wounds can increase the risk of misinterpreting colonization as a true infection. This study had a high percentage of patient-directed discharges, potentially influencing the 30-day rehospitalization rate, and may not accurately reflect the ability to treat xylazine-associated wound infections effectively. The total duration of therapy may be overestimated due to the inability to accurately assess the patient's compliance to oral antimicrobial therapy prescribed upon discharge. Nevertheless, all the aforementioned limitations highlight the many challenges clinicians encounter in real-world clinical practice when treating such wounds.

## CONCLUSIONS

More than half of patients with suspected infected xylazine-associated wound infections had clinical microbiology results to guide antimicrobial therapy. Gram-positive organisms were most frequently isolated, while gram-negative organisms were infrequently observed. These observations suggest that empiric antimicrobial coverage for suspected infected xylazine-associated wound infections should include directed MRSA and β-hemolytic streptococci coverage. Empiric anti-pseudomonal gram-negative and/or anaerobic coverage may not be necessary for most patients being treated for SSTIs, although it may be warranted among those with higher suspicion for bone and joint involvement. Avoiding routine culturing of chronic wounds can minimize misinterpreting colonization as an acute infection.

## Supplementary Material

ofaf384_Supplementary_Data

## Supplementary Data

Supplementary materials are available at *Open Forum Infectious Diseases* online. Consisting of data provided by the authors to benefit the reader, the posted materials are not copyedited and are the sole responsibility of the authors, so questions or comments should be addressed to the corresponding author.

## Notes

***Author contributions.*** M. H. conceptualized the study. D. M. B., with input from C. M., A. B., S. D. F., S. C. H., M. H., and D. P., designed the study. D. M. B. conducted the literature review, performed all chart reviews and statistical analyses, and wrote the first draft of the manuscript. All authors contributed to data interpretation and critical revisions of the report. All authors assume responsibility for the validity and accuracy of the data.

***Acknowledgments.*** We would like to thank all the healthcare professionals and support staff involved in the care of these patients.

***Data availability.*** Data are available in the Supplementary material.
