# Supplementary material for: Clinical Microbiology in Xylazine-Associated Wound Infections
Source: Open Forum Infect Dis. 2025 Aug 7;12(8):ofaf384. doi: 10.1093/ofid/ofaf384 (PMC12342357; doi:10.1093/ofid/ofaf384)
Supplement: ofaf384_Supplementary_Data [file ofaf384_supplementary_data.pdf]

Supplementary Material

Table of Contents

1. Definitions 2
2. Supplemental Table 1. 4
3. Supplemental Table 2. 5
4. Supplemental Table 3. 6

1. **Definitions**
   a. Suspected infection of xylazine-associated wounds requiring antibiotics.
      i. Defined by documentation from the provider in the electronic health record (EHR).
   b. Documentation at an outside hospital.
      i. Defined as documentation available in the EHR using the "Care Everywhere" function at the time of chart review.
   c. Previous hospitalization within 90 days of the index encounter.
      i. Defined as any hospital stay that was at least 48 hours in length, including hospitalization documented at outside hospitals. The 48-hour threshold was implemented to avoid misleading high hospitalization rates due to frequent patient-directed discharges. Hospitalizations exceeding 48 hours are a cutoff used in the surveillance of hospital-onset infections due to the increased risk of hospital-associated infections caused by methicillin-resistant *Staphylococcus aureus* (MRSA) or *Pseudomonas aeruginosa.*[15]
   d. Number of ED visits/hospitalizations in the past 90 days.
      i. Defined as any hospitalization or emergency department (ED) visit, including hospitalization documented at outside hospitals.
   e. Re-hospitalization for infection-related concerns due to wounds or undifferentiated sepsis within 30 days post-discharge.
      i. Defined by documentation of suspected infected wounds by the provider in addition to starting antibiotics for at least 48 hours.
   f. Antimicrobial use for at least 48 hours in the last 90 days.
      i. Defined as any antimicrobial regimen administered for at least two consecutive days, including administrations documented at outside hospitals or received an outpatient prescription.
   g. Positive xylazine in the past year.
      i. Defined by a positive urine xylazine test one-year from the encounter.
   h. Patient-reported xylazine-laced drug use.
      i. Defined by documentation in the index encounter.
   i. Terms used to describe xylazine use (i.e., Tranq, Philly dope, etc.).

i. Defined by documentation in the index encounter.

j. Housing insecurity.

i. Defined by documentation in the EHR in the index encounter.

k. Confirmed or suspected invasive infection.

i. Defined by documentation by the provider in the index encounter and categorized as either bacteremia or bone or joint infection.

l. Wound cultures obtained.

i. Defined as the first most invasive culture results available. For example, if a bedside wound swab was obtained on day one, and the patient went to the operating room (OR) for debridement on day three, the OR cultures were recorded; if the patient had multiple OR cultures from multiple debridement procedures during the index encounter, only the first OR culture was recorded.

m. Prevalence of an extended-spectrum β-lactamase (ESBL) producing E*nterobacterales.*

i. Defined as an organism with resistance to ceftriaxone.

n. Anti-pseudomonal coverage.

i. Defined as coverage against *Pseudomonas*, including aztreonam, cefepime, ceftazidime, ceftazidime/avibactam, ceftolozane/tazobactam, ciprofloxacin, imipenem, levofloxacin, meropenem, piperacillin/tazobactam.

o. ESBL coverage.

i. Defined as coverage against ESBL-producing bacteria, including eravacycline, ertapenem, omadacycline, ceftolozane-tazobactam, ceftazidime-avibactam, imipenem, meropenem, tigecycline, fluoroquinolones.

p. Anaerobic coverage.

i. Defined as ampicillin/sulbactam, amoxicillin/clavulanate, clindamycin, metronidazole, piperacillin/tazobactam, meropenem, imipenem, ertapenem.

1. Supplemental Table 1.

Supplemental Table 1. Empiric antimicrobial coverage utilized within the first 24 hours for xylazine-associated wounds

Variable	Total (N=166)
**Anti-MRSA coverage**	165 (99.4)
**History of MRSA within the past six months**	29 (17.5)
**Gram-negative coverage**	95 (57.2)
**Anti-pseudomonal**	81 (48.8)
**ESBL**	-
**Anaerobic coverage**	57 (34.3)

Note. Data is reported as no. (%), unless otherwise indicated. MRSA, methicillin-resistant *Staphylococcus aureus*; ESBL, extended-spectrum β-lactamase.

2. Supplemental Table 2.

Supplemental Table 2. Enterobacterales isolated from patients with xylazine-associated wounds

	Wound Cultures			
**Variable**	**Bedside/Operative** (n=81)	**Bedside** (n=49)	**Operative** (n=32)	**Blood** (n=153)
**Enterobacterales[a]**	**10 (12.3)**	**5 (10.2)**	**5 (15.6)**	**1 (0.7)**
Proteus species	*7 (8.6)*	*3 (6.1)*	*4 (12.5)*	-
Providencia stuartii	*3 (3.7)*	*2 (4.1)*	*1 (3.1)*	-
Morganella morganii	*3 (3.7)*	*1 (2.0)*	*2 (6.3)*	-
Escherichia coli	*2 (2.5)*	*1 (2.0)*	*1 (3.1)*	-
**ESBL-producing isolates**	*1 (1.2)*	*1 (2.0)*	-	-
Providencia rettgeri	*1 (1.2)*	*1 (2.0)*	-	*1 (0.7)*

Note. Data is reported as no. (%), unless otherwise indicated. Operative cultures included both the operating room and investigational radiology. ESBL, extended-spectrum β-lactamase.

Staphylococcus aureus; CoNS, Coagulase-negative staphylococci.

[a] Organisms recovered are not mutually exclusive.

3. Supplemental Table 3.

Supplemental Table 3. Summary of microbiology results from all included patients

						Gram-Positive Pathogens in Wound												Gram-Negative Pathogens in Wound										Pathogens Isolated in Blood									
ID	Suspected or confirmed BJI	Surgical debridement of wounds	Wound cultures obtained	Method of obtaining culture	No Growth	MSSA	MRSA	CoNS	Viridans group streptococci	β-hemolytic streptococci	Other *Streptococcus* species	*Enterococcus faecalis*	*Enterococcus faecium*	*Cutibacterium* species	Gram-positive anaerobe	Other	Unidentifiable	*Pseudomonas aeruginosa*	*Stenotrophomonas maltophilia*	Gram-negative anaerobe	Other	Unidentifiable	*Escherichia coli*	*Morganella morganii*	*Proteus* species	*Providencia rettgeri*	*Providencia stuartii*	MSSA	MRSA	CoNS	β-hemolytic streptococci	Other *Streptococcus* species	Gram-positive anaerobe	Other Gram-positive organisms	Enterobacterales	Gram-negative anaerobe	Candida species
**1**	X		X	B			X																		X												
**2**	X		X	B						X					X										X		X		X								
**3**	X		X	B			X			X																			X								
**4**	X		X	B			X																								X						
**5**	X		X	B			X																						X		X						
**6**	X		X	B						X								X								X				X							
**7**			X	B			X																														
**8**			X	B	X																																
**9**			X	B			X			X																											
**10**	X		X	B											X									X	X												
**11**			X	B			X																														
**12**			X	B			X		X																												
**13**			X	B			X			X																											
**14**	X		X	B						X																					X						
**15**			X	B			X																														
**16**			X	B																		X															

						Gram-Positive Pathogens in Wound												Gram-Negative Pathogens in Wound										Pathogens Isolated in Blood									
ID	Suspected or confirmed BJI	Surgical debridement of wounds	Wound cultures obtained	Method of obtaining culture	No Growth	MSSA	MRSA	CoNS	Viridans group streptococci	β-hemolytic streptococci	Other *Streptococcus* species	*Enterococcus faecalis*	*Enterococcus faecium*	*Cutibacterium* species	Gram-positive anaerobe	Other	Unidentifiable	*Pseudomonas aeruginosa*	*Stenotrophomonas maltophilia*	Gram-negative anaerobe	Other	Unidentifiable	*Escherichia coli*	*Morganella morganii*	*Proteus* species	*Providencia rettgeri*	*Providencia stuartii*	MSSA	MRSA	CoNS	β-hemolytic streptococci	Other *Streptococcus* species	Gram-positive anaerobe	Other Gram-positive organisms	Enterobacterales	Gram-negative anaerobe	Candida species
17			X	B			X			X																				X							
18			X	B				X																													
19			X	B						X																											
20			X	B			X																														
21	X		X	B			X																														
22			X	B			X			X																											
23			X	B		X																															
24			X	B			X																														
25			X	B						X																											
26			X	B				X		X																											
27			X	B			X																														
28			X	B						X																											
29			X	B			X																														
30			X	B			X			X																											
31			X	B				X																													
32			X	B			X																														
33			X	B			X																														
34			X	B			X																														
35			X	B			X																														
36			X	B			X																														

						Gram-Positive Pathogens in Wound												Gram-Negative Pathogens in Wound										Pathogens Isolated in Blood									
ID	Suspected or confirmed BJI	Surgical debridement of wounds	Wound cultures obtained	Method of obtaining culture	No Growth	MSSA	MRSA	CoNS	Viridans group streptococci	β-hemolytic streptococci	Other *Streptococcus* species	*Enterococcus faecalis*	*Enterococcus faecium*	*Cutibacterium* species	Gram-positive anaerobe	Other	Unidentifiable	*Pseudomonas aeruginosa*	*Stenotrophomonas maltophilia*	Gram-negative anaerobe	Other	Unidentifiable	*Escherichia coli*	*Morganella morganii*	*Proteus* species	*Providencia rettgeri*	*Providencia stuartii*	MSSA	MRSA	CoNS	β-hemolytic streptococci	Other *Streptococcus* species	Gram-positive anaerobe	Other Gram-positive organisms	Enterobacterales	Gram-negative anaerobe	Candida species
37			X	B			X																														
38			X	B			X				X																		X								
39			X	B			X																														
40			X	B				X																				X			X						
41			X	B						X																											
42	X		X	B			X																														
43			X	B			X																														
44			X	B			X																														
45			X	B			X																														
46			X	B		X									X																						
47			X	B				X																													
48			X	B																			X														
49			X	B			X																														
50	X		X	IR		X				X																		X									X
51	X	X	X	IR			X																														
52	X		X	IR			X																						X								
53			X	IR			X																														
54			X	IR			X																						X								
55	X	X	X	OR														X						X					X								
56		X	X	OR		X		X																													

						Gram-Positive Pathogens in Wound												Gram-Negative Pathogens in Wound										Pathogens Isolated in Blood									
**ID**	**Suspected or confirmed BJI**	**Surgical debridement of wounds**	**Wound cultures obtained**	**Method of obtaining culture**	**No Growth**	**MSSA**	**MRSA**	**CoNS**	**Viridans group streptococci**	**β-hemolytic streptococci**	**Other *Streptococcus* species**	***Enterococcus faecalis***	***Enterococcus faecium***	***Cutibacterium* species**	**Gram-positive anaerobe**	**Other**	**Unidentifiable**	***Pseudomonas aeruginosa***	***Stenotrophomonas maltophilia***	**Gram-negative anaerobe**	**Other**	**Unidentifiable**	***Escherichia coli***	***Morganella morganii***	***Proteus* species**	***Providencia rettgeri***	***Providencia stuartii***	**MSSA**	**MRSA**	**CoNS**	**β-hemolytic streptococci**	**Other *Streptococcus* species**	**Gram-positive anaerobe**	**Other Gram-positive organisms**	**Enterobacterales**	**Gram-negative anaerobe**	**Candida species**
**57**	X	X	X	OR			X																														
**58**		X	X	OR						X		X						X		X												X					
**59**	X	X	X	OR													X	X		X								X									
**60**	X	X	X	OR											X																X						
**61**	X	X	X	OR			X																						X								
**62**	X	X	X	OR				X		X																											
**63**		X	X	OR			X																						X								
**64**	X	X	X	OR								X						X																			
**65**	X	X	X	OR			X								X																						
**66**	X	X	X	OR		X				X								X	X									X									
**67**		X	X	OR						X																											
**68**		X	X	OR																X					X												
**69**		X	X	OR			X											X		X	X		X		X		X		X								
**70**	X	X	X	OR														X																			
**71**		X	X	OR			X			X																			X								
**72**	X	X	X	OR			X			X			X																								
**73**		X	X	OR			X									X				X	X			X	X									X			
**74**	X	X	X	OR			X					X								X																	
**75**		X	X	OR	X																																
**76**			X	OR						X																											

						Gram-Positive Pathogens in Wound												Gram-Negative Pathogens in Wound										Pathogens Isolated in Blood									
**ID**	**Suspected or confirmed BJI**	**Surgical debridement of wounds**	**Wound cultures obtained**	**Method of obtaining culture**	**No Growth**	**MSSA**	**MRSA**	**CoNS**	**Viridans group streptococci**	**β-hemolytic streptococci**	**Other *Streptococcus* species**	***Enterococcus faecalis***	***Enterococcus faecium***	***Cutibacterium* species**	**Gram-positive anaerobe**	**Other**	**Unidentifiable**	***Pseudomonas aeruginosa***	***Stenotrophomonas maltophilia***	**Gram-negative anaerobe**	**Other**	**Unidentifiable**	***Escherichia coli***	***Morganella morganii***	***Proteus* species**	***Providencia rettgeri***	***Providencia stuartii***	**MSSA**	**MRSA**	**CoNS**	**β-hemolytic streptococci**	**Other *Strepotococus* species**	**Gram-positive anaerobe**	**Other Gram-positive organisms**	**Enterobacterales**	**Gram-negative anaerobe**	**Candida species**
**77**			X	OR			X																						X								
**78**	X	X	X	OR			X																		X										X		
**79**		X	X	OR										X																							
**80**	X	X	X	OR	X																																
**81**		X	X	OR						X																											
**82**																															X						
**83**		X																																		X	
**84**	X																											X									
**85**	X																												X								
**86**	X																																X				
**87**																															X						
**88**																															X						
**89**																													X								
**90**																												X									
**91**																															X						
**92**																															X						
**93**																													X								
**94**	X																													X							
**95**																													X								
**96**	X																														X						

Note. BJI: Bone or joint infection; Method wound cultures were obtained: B: Bedside; IR: Investigational radiology; OR: Operating room. MSSA: Methicillin-susceptible *Staphylococcus aureus*; MRSA: Methicillin-resistant *Staphylococcus aureus*; CoNS: Coagulase-negative *Staphylococcus*.
